# Mechanical properties of symmetric and asymmetric DNA A-tracts: implications for looping and nucleosome positioning

**DOI:** 10.1093/nar/gku338

**Published:** 2014-05-28

**Authors:** Tomáš Dršata, Naďa Špačková, Petr Jurečka, Marie Zgarbová, Jiří Šponer, Filip Lankaš

**Affiliations:** 1 Institute of Organic Chemistry and Biochemistry, Academy of Sciences of the Czech Republic, Flemingovo nám. 2, 16610 Prague, Czech Republic; 2 Institute of Biophysics, Academy of Sciences of the Czech Republic, Královopolská 135, 61265 Brno, Czech Republic; 3 Regional Centre of Advanced Technologies and Materials, Department of Physical Chemistry, Faculty of Science, Palacký University, 17. listopadu 12, 77146 Olomouc, Czech Republic; 4 CEITEC–Central European Institute of Technology, Campus Bohunice, Kamenice 5, 62500 Brno, Czech Republic

## Abstract

A-tracts are functionally important DNA sequences which induce helix bending and have peculiar structural properties. While A-tract structure has been qualitatively well characterized, their mechanical properties remain controversial. A-tracts appear structurally rigid and resist nucleosome formation, but seem flexible in DNA looping. In this work, we investigate mechanical properties of symmetric A_n_T_n_ and asymmetric A_2n_ tracts for *n* = 3, 4, 5 using two types of coarse-grained models. The first model represents DNA as an ensemble of interacting rigid bases with non-local quadratic deformation energy, the second one treats DNA as an anisotropically bendable and twistable elastic rod. Parameters for both models are inferred from microsecond long, atomic-resolution molecular dynamics simulations. We find that asymmetric A-tracts are more rigid than the control G/C-rich sequence in localized distortions relevant for nucleosome formation, but are more flexible in global bending and twisting relevant for looping. The symmetric tracts, in contrast, are more rigid than asymmetric tracts and the control, both locally and globally. Our results can reconcile the contradictory stiffness data on A-tracts and suggest symmetric A-tracts to be more efficient in nucleosome exclusion than the asymmetric ones. This would open a new possibility of gene expression manipulation using A-tracts.

## INTRODUCTION

A-tracts are commonly defined as DNA sequences of at least four consecutive A-T base pairs without an intervening TA step. When embedded in a general sequence, they bend the DNA double helix towards the minor groove in the centre of the A-tract. The bending magnitude depends on the A-tract length and sequence as well as on temperature and ionic composition of the buffer. A-tracts exhibit a particular structure characterized by large negative propeller and a minor groove progressively narrowing in the 5′ to 3′ direction of the adenine strand. They also have a spine of hydration in the minor groove. This unique structure is formed cooperatively when passing from three to four A-T pairs in a row. At elevated temperatures, A-tracts undergo a cooperative pre-melting transition in which their unique conformation is transformed into a more B-DNA-like structure. Properties of A-tracts and their prominent importance in DNA biology and biophysics have been reviewed multiple times (1–6). In particular, A-tracts affect nucleosome positioning which, in turn, is critical for regulating gene expression (7,8). By manipulating A-tracts, gene expression can be tuned in a predictable manner (9,10).

While structural features of A-tracts seem to be qualitatively well established, their mechanical properties are not fully understood. Crystallographic studies suggest that A-tracts are conformationally rigid. Although the crystallized oligomers are bent in very different directions dictated by crystal packing, the A-tracts themselves are almost straight and have similar conformations (4,11,12). Early molecular dynamics (MD) simulations also indicated a rigid A-tract (13–15). More recent simulation studies report that fluctuations of twist, rise and minor groove width in the A-tract are always smaller than in the flanking sequences and do not change with imposed external bending (16,17). Nuclear magnetic resonance (NMR) carbon spin relaxation combined with MD simulations suggest exceptionally small sugar flexibility within A-tracts (18). Another recent MD work (19) has found that water bridge occupancies in the spine of hydration correlate with DNA stiffness assessed using the standard local dinucleotide model (20–23). Consistent with the view of a ridig A-tract, it has been shown that TATA boxes containing 3–4 consecutive adenines are best described as context-independent structures conforming to a nearest-neighbour non-additive model of protein binding (24). Imino proton resonance measurements show that A-T base pairs within A-tracts have lifetimes at least an order of magnitude longer than in other B-DNA duplexes (25).

However, A-tracts may not be exceptionally rigid in all circumstances. Cyclization kinetics data suggest that the A_4_ sequence is not more rigid, both in bending and in twisting, than many other tetranucleotide sequences (26). Another cyclization study has found bending rigidity of a sequence with phased A_6_ tracts to be similar to that of generic DNA (27). In fluorescence-based, protein-free cyclization experiments (28), inserting A_n_ tracts in the middle of a random non-A-tract sequence resulted in prolongation of looping times, but for some tracts (n = 10, 17), these times were shorter than looping times of another non-A-tract sequence. A very recent work reports that A_n_ tracts appear highly flexible in the context of transcription factor-mediated DNA looping (29).

Another open question concerns properties of the symmetric A_n_T_n_ tracts compared to asymmetric, homopolymeric A_2n_ tracts of the same length. It was discovered early on that phased A_3_T_3_ and A_4_T_4_ tracts migrate anomalously slowly in gel electrophoresis (30,31). Gel mobilities of phased A_4_T_4_ were found to be similar to the properties of A_6_ (32). A later study reported a slightly lower bending induced by A_3_T_3_ compared to A_6_ in the same sequence context, but the difference was judged to be only marginally significant (33). Another work also found phased A_4_T_4_ to migrate anomalously slowly, albeit faster than A_6_ (34). Just like A_2n_ tracts, A_n_T_n_ have exceptionally long base pair lifetimes (25). Crystal structures (35,36), NMR data (34) and molecular modelling (6,13,37,38) also suggest that symmetric and asymmetric tracts share similar basic characteristics. On the other hand, the two A-tract types can hardly be entirely identical, due to the palindromic sequence and the presence of an AT step in the symmetric tract. Indeed, it has been proposed that the structural basis of bending induced by symmetric and asymmetric tracts may differ (35).

**Figure 1.**
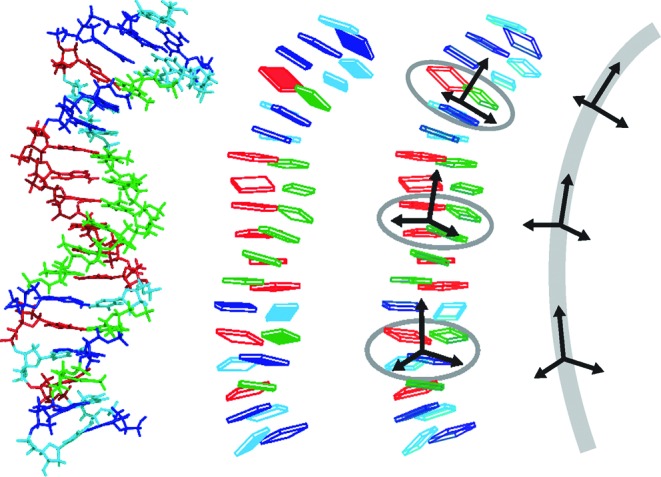
The multiscale modelling approach used in this work. Structures from all-atom MD (left) are used to infer parameters of a non-local rigid base model. Averaging base-fixed frames of selected bases yields mean frames which, in turn, define a description of the DNA oligomer as a piece of anisotropically bendable and twistable elastic rod (right). The rigid base diagrams were produced with 3DNA.

In this work we set out to investigate structure and mechanical properties of A-tracts using molecular modelling. We focus on A_3_T_3_, A_4_T_4_ and A_5_T_5_ symmetric tracts and their asymmetric counterparts A_6_, A_8_ and A_10_, all embedded in a non-A-tract sequence. To describe their properties, we use two types of coarse-grained DNA models (Figure 1).

The first model represents DNA as an ensemble of interacting rigid bases with an underlying quadratic, non-local deformation energy function (22,23,39–41). Every base can in principle be coupled to any other base in the oligomer, yielding a full description of base–base interactions in the harmonic approximation. This substantially extends the standard local models in which individual dinucleotide steps (20,21) or individual base pairs (42,43) are characterized by 6D quadratic deformation energies. The local models have already been successful in problems, such as sequence-dependent nucleosome positioning, identifying transcription factor binding sites and promoter location, or unravelling basic mechanical features of DNA (44–57). Nevertheless, the local models necessarily miss conformational couplings between intra-basepair and inter-basepair coordinates, some of which are well documented (58). They also neglect all couplings between different base pairs or steps. These long-range interactions are expected to be particularly important in A-tracts with their delocalized, interlocked structure, and are properly accounted for by the non-local description used here.

The second model we employ in this work represents a whole DNA fragment as an elastic rod with anisotropic bending and twisting rigidities. It is close in spirit to the description already proposed some years ago (59). Parameters for both the rigid base and the elastic rod models are consistently deduced from extensive, state-of-the-art atomic-resolution MD simulations with explicit inclusion of water and ions. We compare detailed shape and stiffness properties of the A-tracts, and discuss their implications. By threading the A-tracts through a nucleosome structure, we demonstrate the difference between symmetric and asymmetric A-tracts with respect to nucleosome positioning. Our results should help to better understand the behaviour of A-tracts in DNA looping and nucleosome positioning. In particular, our data suggest that using A_n_T_n_ tracts instead of A_2n_ tracts may open yet another possibility to modulate nucleosome positioning and thus affect gene regulation.

## MATERIALS AND METHODS

### Model of interacting rigid bases

In this model we describe a DNA oligomer as an ensemble of interacting rigid bodies representing individual bases (22,23,39–41). The relative orientation and displacement of the bases is defined by intra-basepair coordinates buckle, propeller, opening, shear, stretch and stagger, and inter-basepair or step coordinates tilt, roll, twist, shift, slide and rise (58,60). For a DNA molecule of *n* base pairs, there are $N = 12n - 6$ coordinates which we write as components of a vector **w**. The base–base interactions are characterized by a deformation energy of general quadratic form

(1) \begin{eqnarray*}
&&E({\bf w}) = \frac{1}{2}\left( {{\bf w} - {\bf \hat w}} \right) \cdot {\bf K}\left( {{\bf w} - {\bf \hat w}} \right) = \nonumber \\ &&\frac{1}{2}\sum\limits_{i,j = 1}^N {{\bf K}_{ij} \left( {{\bf w}_i - {\bf \hat w}_i } \right)} \left( {{\bf w}_j - {\bf \hat w}_j } \right)
\end{eqnarray*}

where ${\bf \hat w}$ is the vector of coordinates defining the energy minimum (shape parameters) and **K** is the stiffness matrix. Thus, $E\left( {\bf w} \right)$ is the energy required to distort the coordinates **w** away from their equilibrium values ${\bf \hat w}$. The model is considered to be in contact with a thermal bath of temperature *T*. Assuming small fluctuations of the coordinates, their probability distribution can be well approximated by an *N*-dimensional Gaussian. Consequently, the model parameters ${\bf \hat w}$ and **K** obey the relations

(2) \begin{equation*} {\bf \hat w} = \left\langle {\bf w} \right\rangle,\quad {\bf K} = k_B T{\bf C}^{ - 1} \end{equation*}

where $\left\langle {\bf w} \right\rangle$ is the vector of coordinate means, **C** is the covariance matrix of the coordinates, *k_B_* is the Boltzmann constant and the superscript −1 denotes the matrix inverse.

The stiffness matrix contains all the information about the oligomer deformability (or flexibility) described by the model. Besides that, it is also desirable to characterize the flexibility in an overall manner by one simple parameter. To this end we propose to use the conformational entropy (61), defined by the Gibbs formula

(3) \begin{equation*} S_c = - k_B \int {p({\bf w})\ln } p({\bf w})d{\bf w} \end{equation*}

Substituting the *N-*dimensional Gaussian distribution for $p\left( {\bf w} \right)$ in Equation (3), we find

(4) \begin{equation*} S_c = \frac{1}{2}k_B \ln \left[ {(2\pi e)^N \det {\bf C}} \right] \end{equation*}

where *e* = 2.718 … is the base of the natural logarithm and det denotes the matrix determinant. The entropy *S_c_*, however, is an extensive quantity, meaning that it will increase with the number of rigid base coordinates (i.e. with the oligomer length). It is more convenient to work with entropy per coordinate, given by

(5) \begin{equation*} s_c = S_c/N \end{equation*}

which is intensive and, therefore, enables one to compare oligomers of different lengths.

The deformation may be specified by prescribing only some of the coordinates, while the others are unconstrained and free to relax to their energetically optimal values. For instance, base pair step coordinates only, or even a subset thereof, are often considered. To describe this situation, let us write the coordinate vector **w** as ${\bf w} = ({\bf w}_A,{\bf w}_B )$, where only **w***_A_* are prescribed and ${\bf w}_B$are left unconstrained. The energy associated with deforming the coordinates **w***_A_* is obtained by minimizing the deformation energy with respect to ${\bf w}_B$,

(6) \begin{equation*} \tilde E({\bf w}_A ) = \mathop {\min }\limits_{{\bf w}_{\bf B} } E({\bf w}). \end{equation*}

Performing the minimization, we deduce that

(7) \begin{equation*} \tilde E({\bf w}_A ) = \frac{1}{2}\left( {{\bf w}_A - {\bf \hat w}_A } \right) \cdot {\bf \tilde K}\left( {{\bf w}_A - {\bf \hat w}_A } \right) \end{equation*}

where

(8) \begin{equation*} {\bf \hat w}_A = \left\langle {{\bf w}_A } \right\rangle,\quad {\bf \tilde K} = k_B T{\bf \tilde C}^{ - 1} \end{equation*}

and ${\bf \tilde C}$ is the covariance matrix of the coordinates ${\bf w}_A$. Thus, Equations (7) and (8) of this partially relaxed model are exactly analogous to Equations (1) and (2), except that only a subset of the coordinates **w**, namely **w***_A_*, is now involved.

In particular, if **w***_A_* contains only one coordinate, say twist $\omega _a$ of the base pair step *a*, then Equation (8b) reduces to

(9) \begin{equation*} K_a = \frac{{k_B T}}{{\left\langle {\left( {\omega _a - \left\langle {\omega _a } \right\rangle } \right)^2 } \right\rangle }} \end{equation*}

and gives the force constant $K_a$ associated with imposing an excess twist to step *a*, with all the other coordinates in the oligomer unconstrained. The quantity in the denominator is the variance (or square SD) of $\omega _a$.

Finally, let **w***_A_* contain the six base pair step coordinates of step *a*. Equations (7) and (8) are then just the usual relations for the local dinucleotide model. The meaning of the 6 × 6 dinucleotide stiffness matrix, reported by various authors, is now clear: it is the stiffness matrix associated with a deformation where only the coordinates of that particular base pair step are prescribed, while all the other coordinates in the oligomer remain unconstrained.

### Elastic rod model

In this coarser description, the conformation of the whole oligomer is characterized by just three coordinate frames, two of them located at its ends and one in the middle (Figure 1). The *x*-axis of the middle frame points to the major groove in the centre of the oligomer. Contrary to an earlier similar approach (59), we do not rely on the optimal curvilinear helical axis, since it may introduce artefacts to elastic properties (62). Instead, we obtain the coordinate frames by averaging base-fixed standard reference frames at the appropriate locations (Figure 1). The magnitude $\vartheta$ of bending is the angle between the *z*-axes of the two end frames, the bending direction $\varphi$ is measured with respect to the major groove in the oligomer centre, with $\varphi = 0$ for bending towards the major groove and $\varphi = \pi$ (or 180°) for bending towards the minor groove. The procedure was described in detail earlier (6). Besides $\vartheta$ and $\varphi$, it will be convenient here to introduce the quantities $\rho$ and $\tau$ (which we call global roll and global tilt, respectively) by the relations

(10) \begin{equation*} \rho = \vartheta \cos \varphi,\quad \tau = \vartheta \sin \varphi \end{equation*}

To complement the structural description, we introduce the total twist $\omega$, computed as the sum of local dinucleotide twists between the end frames (half the values are taken at end steps). Assuming a quadratic deformation energy and small fluctuations of the coordinates $\rho$, $\tau$ and $\omega$, we can adapt the formalism of Equations (1) and (2). Stiffness constants per unit length have to be introduced to obtain length-independent material properties. It is also convenient to express the stiffness constants in units of length, in analogy with persistence length of semi-flexible polymers. The deformation energy then takes the form

(11) \begin{equation*} E_r ({\bf u}) = \frac{{k_B T}}{{2l_0 }}\left( {{\bf u} - {\bf \hat u}} \right) \cdot {\bf K}_r \left( {{\bf u} - {\bf \hat u}} \right) \end{equation*}

where ${\bf u} = \left( {\rho,\tau,\omega } \right)$ is the vector of coordinates (in radians), ${\bf \hat u}$ is the coordinate vector specifying the energy minimum, ${\bf K}_r$ the 3 × 3 stiffness matrix and $l_0$ the equilibrium length of the oligomer. The length $l_0$ is computed as the sum of local rises (half the values are taken at end steps), averaged over the MD trajectory. The model parameters ${\bf \hat u}$ and ${\bf K}_r$ are inferred from the fluctuations of **u** using the relations exactly analogous to Equation (2).

### Sequence design, MD simulations and model parametrization

**Figure 2.**
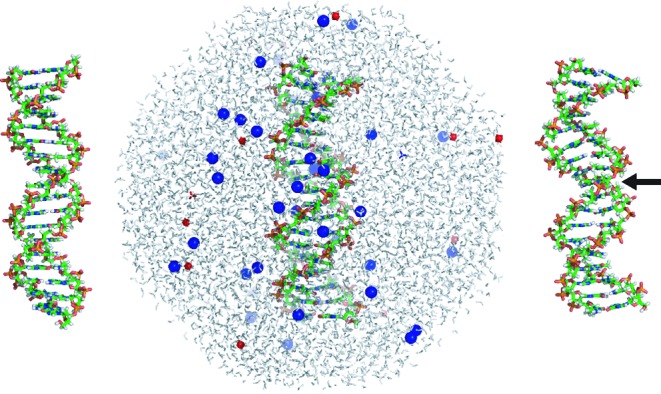
Example of the simulated system. For each simulation, an 18-bp oligomer in canonical B-DNA conformation (left) was chosen as the starting structure. It was solvated by water molecules in an octahedral periodic box, with K^+^ and Cl^−^ ions added to neutralize the DNA charge and mimic the physiological salt concentration of 150 mM KCl (middle). The final structure of the A_3_T_3_ oligomer after one microsecond of MD (right) clearly shows bending towards the minor groove in its centre (marked by the arrow).

Seven 18-base pair (bp) DNA sequences have been investigated. The control C/G-rich sequence comes from the chicken β^A^-globin promoter and was used earlier in nucleosome positioning studies (63,64). We then modified the control sequence to include in its centre the A_3_T_3_, A_4_T_4_ and A_5_T_5_ symmetric tracts, and their asymmetric counterparts A_6_, A_8_ and A_10_. All the tracts are consistently embedded in the 5′-G and C-3′ sequence context. The list of sequences is in Table 1. The oligomers were built as canonical B-DNA and solvated in an octahedral periodic box with extended simple point charge (SPC/E) water, leaving 10 Å between the box walls and the closest DNA atom. In the next step, 34 K^+^ ions were added to neutralize the negative DNA charge, additional 29 K^+^ and 29 Cl^−^ ions were included to mimic the physiological concentration of 150 mM KCl. The ions were parametrized according to Dang (65). Each system contains ca. 10 500 water molecules and 33 000 atoms in total. After equilibration, production runs of unrestrained MD at 300 K and 1 atm were performed using the *parmbsc0* AMBER force field (66). The periodic box volume fluctuates around 334 × 10^3^ Å^3^, the density around 1 g.cm^−3^. The volume occupied by the DNA is about 18 × 10^3^ Å^3^, which represents roughly 5% of the box volume. The *pmemd* module of the AMBER package was used to carry out the simulations. The protocol is very similar to that used by the Ascona B-DNA consortium (67) and is described in detail in Supplementary Methods. The trajectories were extended to 1 microsecond each. Snapshots were recorded every 10 ps and analysed with 3DNA (68) to obtain time series of base-fixed frames, intra-basepair and step coordinates, backbone torsions, minor groove widths, etc. The data then underwent filtering to exclude snapshots with broken intra-basepair hydrogen bonds (cutoff distance 4 Å), as proposed earlier (6,39,47). We tested the effect of filtration and found that its absence would make the stiffness constants less well defined. End base pairs often broke, were not filtered and were ignored in all data processing. The model parameters were computed from Equation (2) where the ensemble averages were replaced by averages over the simulated trajectories. Figure 2 shows the initial canonical B-DNA structure, the simulated system with water and ions, and the final structure of the A_3_T_3_ oligomer after 1 microsecond of simulation.

**Table 1.** Sequences and their properties in the simulations. The underlined pairs include bases defining average frames at the ends and in the middle of the studied sequence (see Materials and Methods and Figure 1). Bending direction of 180° points towards the minor groove in the oligomer centre, 0° indicates bending into the major groove. The errors in parentheses are mean differences between values for the whole trajectory and for its halves.

Sequence	Embedded A-tract	Bending magnitude (°)	Bending direction (°)	Effective isotropic bending stiffness, $a_{iso}$ (nm)	Twisting stiffness, *C* (nm)
GCCTGGAAATTTCTGTGC	A_3_T_3_	12 (0.4)	179 (1)	75 (2)	102 (2)
GCCTGGAAAAAACTGTGC	A_6_	11.9 (0.2)	200 (0)	71 (3)	98 (1)
GCCTGAAAATTTTCGTGC	A_4_T_4_	8.3 (0.0)	178 (3)	82 (2)	119 (1)
GCCTGAAAAAAAACGTGC	A_8_	8.2 (0.2)	212 (2)	70 (2)	101 (0)
GCCGAAAAATTTTTCTGC	A_5_T_5_	2.4 (0.1)	193 (0)	86 (3)	132 (5)
GCCGAAAAAAAAAACTGC	A_10_	6.9 (0.3)	283 (4)	73 (1)	117 (2)
GCCTGGCGCGCGCTGTGC	control	3.5 (0.1)	356 (180)	72 (0)	112 (2)

To analyse the stiffness matrix as a whole (e.g. to compute the entropy), its entries have to be made dimensionally uniform. To this end we non-dimensionalize the coordinates using the length scale 1 Å and angle scale equal to 360/34, or 10.6°, as conventionally done (23,59,69). A simple computation shows that the entropy *S_c_* (Equation (4)) changes with coordinate rescaling, but differences of *S_c_* do not. If one uses just one scaling factor for all the distances and one for all the angles (as done here), then the differences in entropy per coordinate, *s_c_* (Equation (5)), also do not change with coordinate rescaling.

## RESULTS AND DISCUSSION

### Stiffness at the level of rigid bases

It has been well documented that A_n_ tract structure is not uniform, as minor groove gradually narrows in the 5′ to 3′ direction of the adenine strand (4,70). The anomalously long base pair lifetimes emerge from the second 5′ end A-tract base pair and further increase for the third pair (25). At the 3′ end, the values again return to the usual B-DNA properties. Chemical shift experiments indicate a zone of transitional structure about 4 bp on the 5′ side and 2 bp on the 3′ side, with possible slight extension beyond the 3′ end into the non-A-tract sequence (71). A natural question then arises as to whether such gradual buildup of A_n_ tract characteristics applies to stiffness as well.

**Figure 3.**
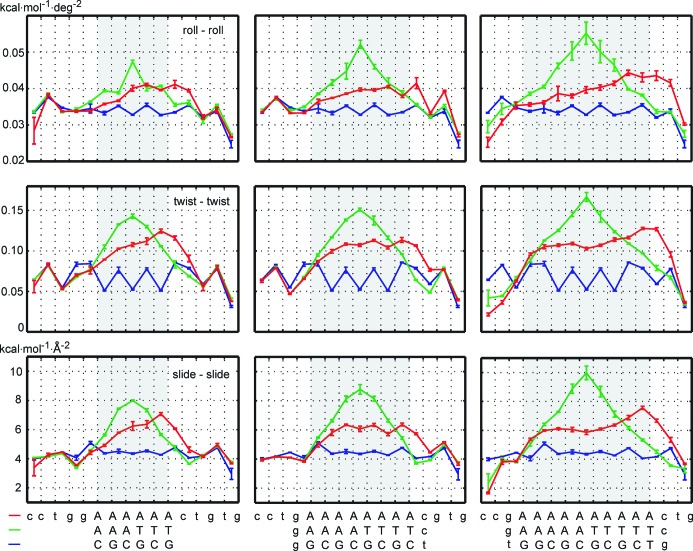
Diagonal stiffness constants of the non-local rigid base model. Stiffness of the asymmetric, homopolymeric A_2n_ tracts (red) builds up gradually in the 5′ to 3′ direction of the adenine strand and eventually reaches a plateau. In contrast, the symmetric A_n_T_n_ tracts (green) have a peak at the central AT step which extends over the whole tract, with no plateau. Moreover, the peak value increases with the tract length. Both A-tract types exhibit higher values than the control sequence (blue). Error bars in this and the other figures are mean differences between values for the whole trajectory and for its halves. This data exemplify the stiffness profiles of A-tracts, complementing the well documented structural profiles. They expose sharp differences between stiffness distribution in symmetric and asymmetric A-tracts.

Figure 3 shows diagonal entries of the rigid base stiffness matrix for roll, twist and slide deformations, data for propeller are in Supplementary Figure S1. The diagonal entries are stiffness constants of a deformation in which the given coordinate is distorted while all the others remain fixed (Equation (1)). For the asymmetric A_n_ tracts, we indeed observe a continuous buildup of stiffness in the 5′ to 3′ direction, a plateau in the middle and return to values of the control sequence at the 3′ end. The transitional regions span 2–3 bp at each end and extend slightly beyond the 3′ end of the tract. The observed extension of the A_n_ tract stiffness beyond its 3′ end (but not its 5′ end) complements the earlier analogous finding about the A_n_ tract structure: A_n_ tracts affect the 3′ flanking sequence, while the 5′ sequence remains unaffected (72). Diagonal stiffness constants for the remaining coordinates (not shown) exhibit the same trend, with the exception of opening and stretch stiffness which are consistently lower and uniform within the A-tract, reflecting just the smaller number of hydrogen bonds in A-T pairs.

Stiffness constants associated with another mode of deformation, namely, the one where a given coordinate is distorted but all the others are free to relax (Equation (9)) follow the same pattern, only the values are now smaller, since the structure is less constrained (Supplementary Figures S1 and S2). The same trend is also observed for the diagonal stiffness constants of the standard local model where the values are between the former two (not shown). Thus, homopolymeric A-tract stiffness follows similar patterns as its structure, reflecting once again a continuous buildup of A_n_ tract properties in its 5′ to 3′ direction.

Stiffness profiles of the symmetric A_n_T_n_ tracts are in striking contrast with the A_2n_ values (Figures 3, Supplementary Figures S1 and S2). The first expected difference stems from the fact that, in contrast to A_2n_, the A_n_T_n_ sequence is palindromic. Therefore (apart from the influence of the flanking sequeces), the stiffness profiles within the A_n_T_n_ tract should be symmetric with respect to its centre (39), which is indeed almost exactly the case. Surprisingly, however, we observe no discernible stiffness plateau within the A_n_T_n_ tracts, not even for A_5_T_5_. The central peak keeps rising with the tract length, suggesting that the limiting value has not yet been reached. This indicates a long-range influence of the central AT step.

The AT step is known to have remarkable properties: it has a sharply defined conformation in one sequence context but varies considerably between contexts (73), and it ranks among the stiffest dinucleotides as judged from the local dinucleotide model (20,21,44,47). This all distinguishes AT from the very flexible TA step (20,21,44,47,73). It has been found earlier that a TA step has only a local effect on surrounding A_n_ tracts, thus T_n_A_n_ are best understood as two adjacent A_n_ blocks with opposite polarity (31). But can A_n_T_n_ also be described just as two consecutive A_n_ tracts? Our stiffness data indicate that they cannot, at least not for $n \le 5$. Obviously, the effect of the central AT step should ultimately vanish upon increasing the tract length. Thus, a very long A_n_T_n_ tract would eventually adopt the poly-A conformation inside its A_n_ blocks, but our data suggest that *n* would have to be much higher than 5.

**Figure 4.**
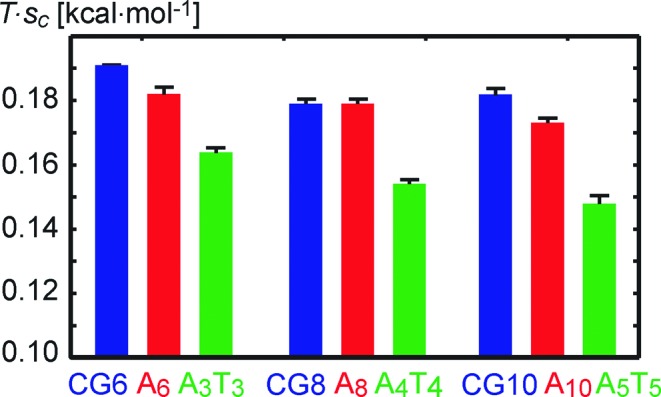
Entropy per rigid base coordinate *s_c_*, multiplied by *T =* 300 K. The symmetric A_2n_ tracts (red) have slightly lower or equal entropy compared to the control sequence (blue). In contrast, the symmetric A_n_T_n_ tracts (green) always have lower entropy (are more rigid) than the asymmetric tracts and the control. Rigidity per coordinate increases as the A-tracts get longer. CG*m* denotes the *m* central pairs of the control sequence.

Apart from special types of deformation, it is desirable to capture the overall stiffness by one simple parameter. The conformational volume has traditionally been used for this purpose (19–21,51,74). For a larger number of coordinates, however, it starts to be very large and not practical to use. Instead, we propose to measure the overall stiffness by the conformational entropy per rigid base coordinate, as defined by Equation (5). Besides its tractable values, this quantity also has the advantage of being intensive, i.e. it enables one to compare oligomers of different lengths. Figure 4 shows entropy per coordinate for the A-tracts and for the corresponding parts of the control sequence. Entropy of the asymmetric A_2n_ tracts is equal or only slightly lower than the control. This indicates the existence of deformation modes, others than those in Figure 3, in which A_2n_ is actually more flexible than the control. These flexible modes manifest themselves in global stiffness properties described below. In contrast, the symmetric A_n_T_n_ tracts are more rigid (have lower entropy) than the control and their A_2n_ counterparts. The entropy per coordinate decreases with A-tract length, meaning that prolonging A-tracts results in their overall rigidification.

### Local structure and backbone dynamics

Equilibrium values of the local coordinates (Supplementary Figure S3) and minor groove widths (Supplementary Figure S4) confirm known structural characteristics of A-tracts. Propeller of A_n_T_n_ clearly reaches a plateau, whereas roll does not, probably reflecting once again the non-local effect of the central AT step. The BII populations (Supplementary Figure S5) are around 10% for the adenine strand and essentially zero for the thymine strand. The secondary peak at the second AA step from the 5′ end is consistent with increased flexibility at that location detected by NMR (18). Sugar puckers (Supplementary Figure S6) are uniformly higher (more south) in the adenine strand than in the thymine strand.

**Figure 5.**
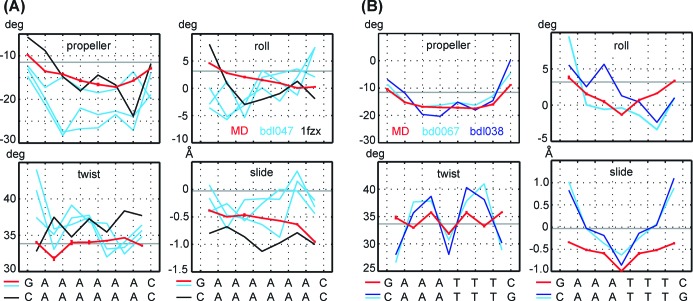
Comparison of equilibrium values of propeller, roll, twist and slide from MD simulations (red) with crystal structures (light and dark blue) and an NMR structure (black). The bdl047 crystal contains three independent structures, which are shown here individually. Generic B-DNA values are represented by horizontal grey lines. All the structures qualitatively capture A-tract properties, notably the high negative propeller. However, there are substantial quantitative differences between the individual experimental structures of A-tracts.

**Figure 6.**
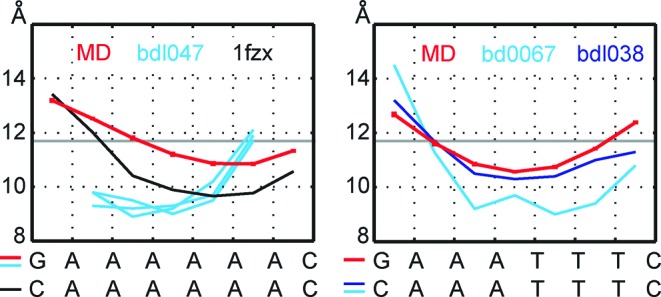
Minor groove profiles from MD simulations and from experimental structures, colour coding as in Figure 5.

It is informative to compare the simulated structures with available crystallographic and NMR data. Figure 5A shows MD values for A_6_ together with data for the three independent structures from a 2.3 Å crystal (bdl047 (12)) and a structure from NMR with residual dipolar couplings (1fzx (75)). Figure 5B compares MD values for A_3_T_3_ with a 2.2 Å and 1.5 Å crystal data (bdl038 (76) and bd0067 (36), respectively). The minor groove widths are in Figure 6. The B-DNA values are also shown in the figures (propeller is taken from (58), roll, twist and slide from (44), minor groove width from canonical B-DNA). All the experimental data show high negative propeller and narrow minor groove, in line with general structural features of A-tracts. However, they are highly variable in their numerical values. For instance, propeller of a given pair may span the range of 13° among different structures, the range of roll may attain 6°. Moreover, the crystallographic roll in Figure 5B is not symmetric, although the crystallized sequence is palindromic. Thus, experimental structures of A-tracts may not be as invariable as traditionally thought. This especially concerns propeller, which (together with buckle) has been identified as the most flexible structural parameter (42). MD values lie mostly within the experimental range, although the minor groove narrowing seems to be underestimated and the MD slide in A_3_T_3_ is too negative.

### Global stiffness and bending

Besides the rigid base description, we also model our DNA oligomers at a longer scale as anisotropically bendable and twistable elastic rods (Figure 1). Their configuration is described by three global coordinates: bending angle towards the grooves in the oligomer centre (global roll, $\rho$), bending angle in the perpendicular direction, i.e. towards the backbone (global tilt, $\tau$) and total twist, $\omega$. The model is again parametrized from structural fluctuations in atomistic MD simulations, as described in Materials and Methods. It is characterized by equilibrium values of the three global coordinates and by the 3 × 3 stiffness matrix.

We first focus on the A-tracts as such (without flanking sequences). To start with, we consider a simplified case of pure bending, while total twist is free to relax. This yields an effective 2 × 2 stiffness matrix with respect to global roll and tilt. The diagonal entries are stiffness constants for bending to the grooves ($a_g$) and to the backbone ($a_b$), respectively. The off-diagonal terms are found to be at least an order of magnitude smaller than the diagonal entries. Thus, the global bending energy is to a high precision just a sum of two quadratic terms, expressing contributions from bending to the grooves and to the backbone. It follows from general properties of quadratic forms (see for instance (77)) that the stiffness constant for bending in any other direction lies between $a_g$ and $a_b$.

The two bending stiffness constants are shown in Table 2, together with the effective isotropic constant $a_{iso}$ given by the relation $2/a_{iso} = 1/a_g + 1/a_b$ (78). The $a_{iso}$ values in Table 2 clearly show that, in terms of global bending, the A_2n_ tract is always more flexible than the C/G-rich control sequence. In contrast, the A_n_T_n_ tract is always stiffer than the control (and stiffer than A_2n_). The values of $a_{iso}$ for the control (72–75 nm) are higher than the consensus persistence length of generic DNA (50 nm) which, however, is a property of a very long stretch of random sequence and also contains a contribution from static conformational disorder.

**Table 2.** Global bending and twist stiffness for the A-tracts alone (without flanking sequences) and for the corresponding parts of the control. The average frames used to compute the properties are defined by bases in two consecutive pairs at the beginning, in the middle and at the end of each indicated tract (e.g. AAAAATTTTT). The errors are computed as in Table 1.

Sequence	Groove bending stiffness, $a_g$ (nm)	Backbone bending stiffness, $a_b$ (nm)	Effective isotropic bending stiffness, $a_{iso}$ (nm)	Twisting stiffness, *C* (nm)
A_3_T_3_	65 (2)	137 (1)	88 (2)	118 (1)
A_6_	49 (0)	118 (1)	69 (0)	102 (1)
GC6^a^	56 (2)	112 (2)	75 (1)	117 (2)
A_4_T_4_	91 (1)	94 (3)	92 (1)	135 (1)
A_8_	60 (2)	80 (0)	68 (1)	101 (1)
GC8^a^	68 (2)	77 (1)	72 (1)	106 (1)
A_5_T_5_	114 (7)	75 (3)	90 (4)	150 (5)
A_10_	83 (3)	61 (3)	70 (1)	113 (1)
GC10^a^	84 (2)	67 (1)	74 (0)	115 (1)

^a^GC*m* denotes *m* central base pairs of the control sequence

Another simple deformation we consider is pure twisting, while bending is unconstrained. The twist stiffness constants *c* are in Table 2. Here again we see that A_n_T_n_ is stiffer and A_2n_ more flexible than the control. The twist stiffness constants are comparable to values of ∼100 nm obtained from single molecule manipulation experiments (79–82). Supplementary Table S1 lists the complete 3 × 3 stiffness matrices which enable one to compute the deformation energy for simultaneous bending and twisting according to Equation (11).

When inserted between the flanking sequences, our A-tracts induce bending to the DNA helix which decreases with A-tract length and is about the same in magnitude for A_n_T_n_ and A_2n_ tracts (Table 1). The length dependence agrees with gel mobility data showing that A_n_ tract bending is maximal for $n = 6$ and then decreases with increasing *n* (83). The bending is towards the minor groove in the A-tract centre. The only outlier in these trends is A_10_, gently bent to the backbone. We were unable to find any simple structural reason for this behaviour. The bending magnitude (12° for A_6_ and A_3_T_3_) is lower than the 17°–21° obtained from cyclization experiments (84). However, those experiments were done at 10 mM MgCl_2_ salt concentration (84). There are indications that A_6_ tracts may induce only a 7° bend in the absence of Mg^2+^ (85), and a recent study provides evidence that A-tracts produce essentially no curvature in near-physiological concentration of monovalent ions (86).

Finally, we investigate the global stiffness of A-tracts together with their flanking sequences (12 bp in total, Table 1). This property is just as relevant as the stiffness of the A-tracts themselves, since in some constructs (e.g. in (27)), a series of phased shorter A-tracts interspersed by non-A-tract sequences is used instead of long A-tracts. Analysis exactly analogous to the above yields bending stiffness of sequences with embedded A_n_ very close to the control, in agreement with experiment (27). In contrast, sequences with embedded A_n_T_n_ are stiffer in bending. Similar results are found also for twisting stiffness (Table 1).

We checked the dependence of the results on the choice of the global coordinates. Replacing the average frames (Figure 1) by the base pair step middle frames from 3DNA yields results identical within error estimates. If we choose the step middle frames, local twist and local rise from Curves+ (87) instead, we obtain nearly identical shape and bending stiffness, while twist stiffness is uniformly about 10% higher than in Table 2. Thus, the tested coordinate choices have negligible effect on the global shape and relative stiffness of our sequences.

### DNA looping and nucleosome positioning

A number of studies have reported that A_n_ tracts are relatively depleted of nucleosomes *in vivo*, mostly due to the tracts’ intrinsically lower nucleosome affinity (see the reviews (7,8) and references therein). As an explanation, it has been suggested that A_n_ tracts resist the structural deformations required for nucleosome formation (7). A very recent study on *Schizosaccharomyces pombe* (88) offers a fresh perspective on the issue. It reports that A_n_ tracts affect but do not deplete nucleosomes in *S. pombe* and that they prefer particular rotational positions. As a result, the A-tract fraction in the nucleosome changes in a periodic manner with the distance from the dyad. Thus, although the A_n_ tracts may perturb nucleosome formation, the effect is likely to be more subtle and depend on the position of the tract within the nucleosome. This view is supported by a structural study of nucleosome core particles containing an A_16_ tract (89). The A-tract fragment conforms well to the topology typical for nucleosomal DNA, but the DNA structure is locally distorted and the interactions between the DNA ends and the histone core are destabilized.

To obtain insight into the A-tract behaviour in the nucleosome, we performed threading of the tracts through a high-resolution nucleosome crystal structure (1kx5 (90)). In threading, the tested DNA fragment is constrained to the DNA geometry at a particular location in the nucleosome and the energy needed to deform the fragment is recorded. The fragment is then shifted by 1 bp along the nucleosomal DNA and the calculation is repeated. As a result, one obtains the deformation energy as a function of the fragment position within the nucleosomal DNA.

**Figure 7.**
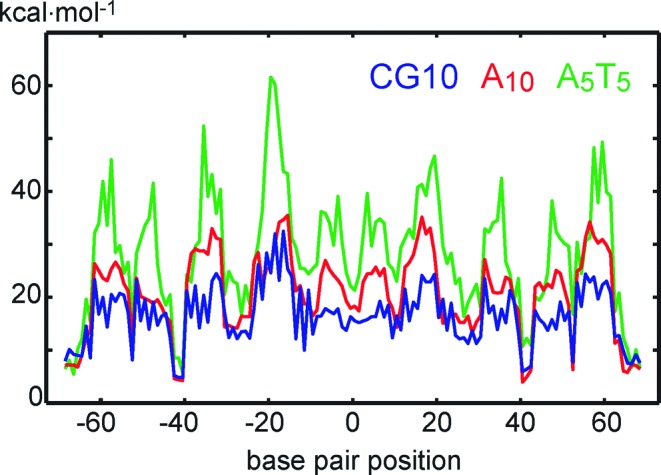
Deformation energy associated with threading of A_10_ and A_5_T_5_ tracts and the central 10 bp of the control sequence (CG10) through the 1kx5 nucleosome structure. Roll, twist and slide are taken from the nucleosomal DNA, all the other coordinates are left unconstrained. The symmetric A_5_T_5_ tract (green) resists deformation more than the asymmetric A_10_ tract (red), whereas the control sequence (blue) is incorporated more easily on average than the two tracts. However, the energy roughly follows a 10-bp periodicity pattern and there are positions where the A_10_ and control values are close to each other, whereas the A_5_T_5_ values are higher at the majority of locations.

In our case, only roll, twist and slide were constrained to the nucleosomal x-ray values, all the other coordinates were left free to relax. This choice is based on the finding that roll, twist and slide are highly conserved among nucleosome structures (91). Thus, we used the partially relaxed model (Equations (7) and (8)) to compute the deformation energy, with the coordinate vector ${\bf w}_A$ containing roll, twist and slide of all the steps. Figure 7 shows deformation energy profiles for threading the A_10_ and A_5_T_5_ tracts compared to GC10, the central 10 bp of the control sequence. Results for the other tracts are analogous. The average deformation energy of A_10_ is 4 kcal/mol higher than average of the control, and it rises by another 7 kcal/mol when passing from A_10_ to A_5_T_5_. These values should be understood as upper bounds, since they do not include full structural adaptation of DNA to nucleosome binding. Although the deformation energy of A_10_ is higher on average than the control, there are locations where the two energies are close to each other. In contrast, the A_5_T_5_ tract is more resistant to deformation (have higher deformation energy) at nearly every position.

The deformation energy in Figure 7 follows roughly a 10-bp periodicity pattern, as recently reported (88). A closer inspection reveals this pattern to correlate with static bending of the A-tracts alone (without flanking sequences), shown in Supplementary Table S2. Note that this is different from the A-tract-induced bending (here reported in Table 1) where A-tracts and the flanking non-A-tract sequences are examined together. All our A-tracts are slightly bent (by 3°–5°) towards the major groove in their centre, thus the energy is lower when the major groove faces the nucleosome core. The A-tract bending direction in experimental structures is inconclusive: the tracts are bent by several degrees to the minor groove (bdl047), major groove (bdl038) or the backbone (bd0067 and 1fzx) (Supplementary Table S2). Threading of A-tracts embedded in their flanking sequences yields more noisy profiles, but with nearly identical phasing as for the tracts themselves (Supplementary Figure S7).

It has been pointed out that DNA sequences behave quite differently with respect to nucleosome positioning and with respect to looping (92). In particular, the asymmetric A_n_ tracts, which may resist nucleosome formation due to their rigidity, are found to be highly flexible in the context of DNA looping (29). We hypothesize that this strikingly different behaviour may be due to a different type of deformation taking place in the two cases. On the one hand, DNA structure in the nucleosome, although flexible to some extent (93), is under local constraint and may be deformed also with respect to translational degrees of freedom such as slide (94). The local displacement of the DNA bases then plays a major role. On the other hand, DNA looping only imposes boundary conditions at the ends and thus probes long length-scale deformation modes. A rather loose definition of the looping boundary conditions, which also involve the protein flexibility (92), adds to the freedom of choosing the most favourable mode.

Our data on the asymmetric A_n_ tracts support this hypothesis. When rigid base coordinates are constrained in the deformation, then A_n_ tracts appear more rigid than the control G/C-rich sequence. We demonstrated this for special types of deformation involving individual coordinates, and by threading the A_n_ tract through the nucleosome. In global bending and twisting, however, A_n_ tracts are more flexible than the control. Note that, just as the rigid base coordinates, the global bending and twisting coordinates again depend only on the relative position and orientation of individual bases. Indeed, the global roll and tilt depend on the end and middle frames which, in turn, are means of selected base-fixed frames, and the total twist is just the sum of local twist values. Thus, both local and global deformations sample the same underlying rigid base energy surface. The difference, however, is that global deformations impose much less constraint on the rigid base displacements and thus enable the system to use a more flexible deformation mode.

Our results for symmetric A_n_T_n_ tracts differ sharply from those for the asymmetric A_2n_ tracts. We find that A_n_T_n_ tracts are stiffer than A_2n_ and the control both at the local and at the global, elastic rod levels. This indicates that A_n_T_n_ tracts should not loop so easily, and should resist nucleosome formation even more than A_2n_ tracts of the same length do.

Recently, artificial insertion of A_n_ tracts of different length and purity into the yeast genome has been used to manipulate nucleosome positioning and thus fine-tune regulation of gene expression (9,10). Our results suggest that by using symmetric A_n_T_n_ tracts instead of asymmetric A_2n_ tracts of the same length, one should be able to obtain an even stronger nucleosome exclusion effect. Equivalently, the same exclusion effect could be obtained with shorter tracts, perturbing less the original genome sequence.

## CONCLUSION

A-tracts are unique structural elements within double-stranded DNA which play a major role in nucleosome positioning and transcription factor binding. Their structural features, such as high negative propeller, narrow minor groove and bending induced to DNA, have been well characterized. In contrast, information about their mechanical properties has been contradictory. A-tracts appear rigid in some circumstances, such as nucleosome formation, but seem flexible in others, notably in DNA looping. To explain these findings, more detailed information about A-tract stiffness is needed but is difficult to obtain experimentally.

In this work we investigate mechanical properties of symmetric (A_n_T_n_) and asymmetric (A_2n_) tracts using a model of rigid bases interacting via a non-local harmonic potential, and another, more global model representing DNA as an anisotropically bendable and twistable elastic rod. We have found that the A-tract stiffness relative to a control G/C-rich sequence depends on the type of deformation they undergo. Asymmetric tracts are stiffer than the control with respect to localized deformations, but more flexible when global bending and twisting takes place. The localized constraints play a role in nucleosome positioning, while global boundary conditions are imposed in looping. Both local and global deformations sample the same underlying base–base deformation energy, but global boundary conditions impose less constraint to base displacements and thus allow the system to use a more flexible deformation mode. Our results, therefore, can reconcile the seemingly contradictory stiffness behaviour of the asymmetric A-tracts.

The symmetric A-tracts are found to be stiffer than the asymmetric ones and stiffer than the control, both with respect to local and global deformation. Thus, our results predict that the symmetric A_n_T_n_ tracts should affect nucleosome formation even more than the asymmetric A_2n_ tracts do. It has already been found that the degree of nucleosome eviction depends on the length of A_n_ tracts and the number of mutations interfering with the A-tract structure, which enables one to fine-tune gene expression by incorporating A_n_ tracts into the genome. Our results open yet another possibility, namely, using the symmetric A_n_T_n_ tracts instead, which are expected to be more efficient in nucleosome exclusion.

In summary, our work establishes detailed stiffness properties of the symmetric A_n_T_n_ and asymmetric A_2n_ DNA A-tracts using coarse-grained models consistently parametrized from large-scale, explicit solvent MD simulations. The results can reconcile the seemingly contradictory stiffness properties of A-tracts, expose the differences in A_n_T_n_ and A_2n_ tract mechanics, and have implications for gene expression manipulation using A-tracts.

## SUPPLEMENTARY DATA

Supplementary Data are available at NAR Online.

SUPPLEMENTARY DATA
