# Supplementary material for: Mechanical properties of symmetric and asymmetric DNA A-tracts: implications for looping and nucleosome positioning
Source: Nucleic Acids Res. 2014 May 28;42(11):7383–94. doi: 10.1093/nar/gku338 (PMC4066768; doi:10.1093/nar/gku338)
Supplement: SUPPLEMENTARY DATA [file supp_42_11_7383__index.html]

Mechanical properties of symmetric and asymmetric DNA A-tracts: implications for looping and nucleosome positioning — Mechanical properties of symmetric and asymmetric DNA A-tracts: implications for looping and nucleosome positioning — Mechanical properties of symmetric and asymmetric DNA A-tracts: implications for looping and nucleosome positioning — SUPPLEMENTARY DATA 

# Mechanical properties of symmetric and asymmetric DNA A-tracts: implications for looping and nucleosome positioning

## SUPPLEMENTARY DATA

**Files in this Data Supplement:**

- SUPPLEMENTARY DATA
