# Supplementary material for: Mechanical properties of symmetric and asymmetric DNA A-tracts: implications for looping and nucleosome positioning
Source: Nucleic Acids Res. 2014 May 28;42(11):7383–94. doi: 10.1093/nar/gku338 (PMC4066768; doi:10.1093/nar/gku338)
Supplement: SUPPLEMENTARY DATA [file supp_gku338_nar-00260-f-2014-File002.pdf]

Mechanical properties of symmetric and asymmetric DNA A-tracts: implications for looping and nucleosome positioning

T. Dršata et al.

Supplementary material

S1 Supplementary methods

S1.1 System preparation

Each of the studied oligomers was built as canonical B-DNA, using the function `bdna()` of the program NAB (part of the Amber Tools 1.0 package). The initial structure together with the force field parameters were then loaded into the program LEaP (Amber Tools 1.0) which enables one to create coordinate and topology files necessary for the simulation.

Some of the hydrogen atoms were missing in the initial structure and they were added automatically by LEaP. To avoid any close contacts of these hydrogens with other atoms, a short energy minimization *in vacuo* was performed using the *sander* module of Amber 10. Positional restraints on all nonhydrogen atoms were imposed (harmonic potential with a 100 kcal.mol^{-1}Å^{-2} force constant). The minimization started with the steepest descent algorithm (250 steps) and then switched to conjugated gradients (250 steps).

Next, we solvated the minimized structure with SPC/E water molecules. LEaP was used to create a periodic box of water molecules in the shape of a truncated octahedron. The minimum distance between any atom of the solute and the edge of the box was chosen to be 10 Å. This resulted in adding ca 10,500 water molecules with the total box volume around 371×10^3 Å^3 and the initial density 0.9 g.cm^{-3}.

The solvated DNA structure was then neutralized with 34 K^+ ions, additional 29 K^+ and 29 Cl^- ions were included to attain a physiological salt concentration of 150 mM. The ions were parameterized according to Dang (ref. (65) in the main text). The LEaP program adds ions to local minima of the electrostatic potential around the solute. To avoid any positional bias, the ion positions were further randomized using the `randomizeions` command of the program *ptraj* (part of Amber Tools 1.0). The predefined minimum distance

between any two ions was chosen to be 3.5 Å and the minimum distance between any ion and the DNA molecule was 5 Å.

This procedure was applied to all the DNA oligomers studied. As a result, we obtained initial configurations of the simulated systems, each with a different DNA oligomer of 18 base pairs, immersed in explicit water and ions at physiological salt concentration. Each of the systems consisted of about 33,000 atoms in total. Prior to the production MD run, all the systems were equilibrated.

S1.2 Equilibration procedure

The equilibration procedure included several energy minimizations and short MD runs with positional restraints of decreasing strength imposed on the DNA. The initial minimization was followed by slow heating, each of the next minimizations was followed by a short equilibration MD run. All the steps were carried out using *sander* module of Amber 10.

The initial minimization consisted of 500 steps of steepest descent followed by 500 steps of conjugated gradients at constant volume periodic boundary conditions, with 25 kcal.mol^{-1}Å^{-2} positional restraints on the DNA. After that, a 100 ps MD run was performed at constant volume with the same positional restraints. During the first 10 ps, the system was heated from 100 K to 300 K and then kept at 300 K. The Berendsen weak-coupling algorithm (coupling constant 0.2 ps) was used for temperature regulation, the nonbonded cutoff distance was set to 9 Å. The bonds which contain hydrogen atoms were constrained using the SHAKE algorithm. The MD time step was 2 ps.

All the subsequent minimizations again comprised 500 steps of steepest descent and 500 steps of conjugated gradients, with decreasing values of positional restraints (5, 4, 3, 2, 1 and 0.5 kcal.mol^{-1}Å^{-2}). Each minimization was followed by a short MD, using the same positional restraints as in the preceding minimization. The MD runs were 50 ps each, carried out at 300 K and 1 atm, with pressure and temperature regulation using the Berendsen algorithm (coupling constants 0.2 ps). The last step of the equilibration phase was another 50 ps MD run at 300 K and 1 atm with no positional restraint and with the Berendsen coupling constants increased to 5.0 ps. The final density fluctuated closely around 1 g.cm^{-3} and the total volume around 334×10^3 Å^3 for all the studied systems.

S1.3 Production

The production phase was performed using the *pmemd* module of Amber 10. Periodic boundary conditions were used, the constant temperature 300 K and pressure 1 atm were maintained by the Berendsen algorithm with coupling constants 5.0 ps, time step was 2 ps. The nonbonded cutoff was set to 9 Å and all the bonds containing hydrogen atoms were treated with the SHAKE algorithm. Translation of the center of mass was removed every 5000 time steps. DNA snapshots were extracted from the simulated trajectories using the *ptraj* module of Amber Tools 1.0.

S2 Supplementary figures and tables

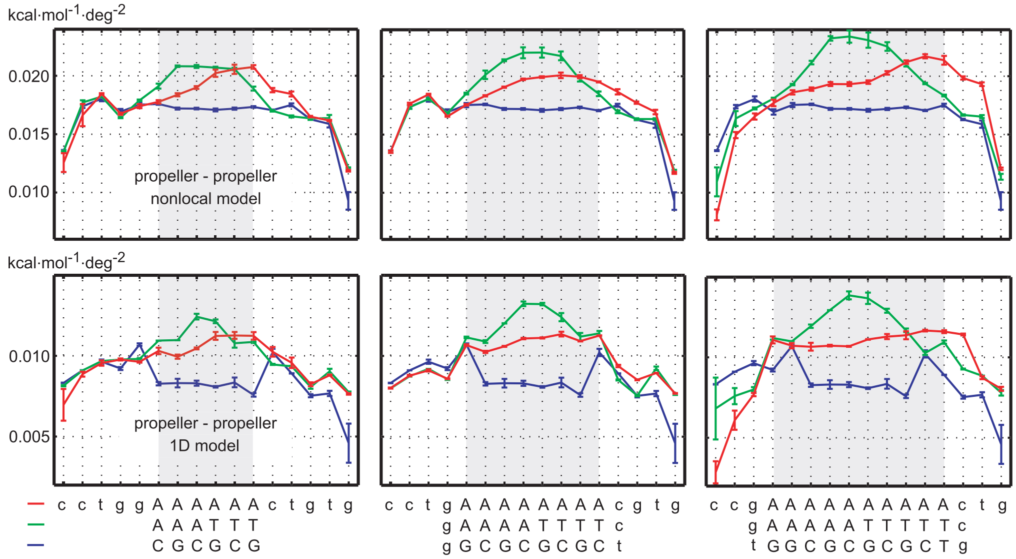

Figure S1: Diagonal stiffness constants related to propeller deformation for the full, non-local rigid base model (upper row) and the unidimensional stiffness constants defined by eq (9) in the main text (lower row). Errors are mean differences between values for the whole trajectory and for its halves.

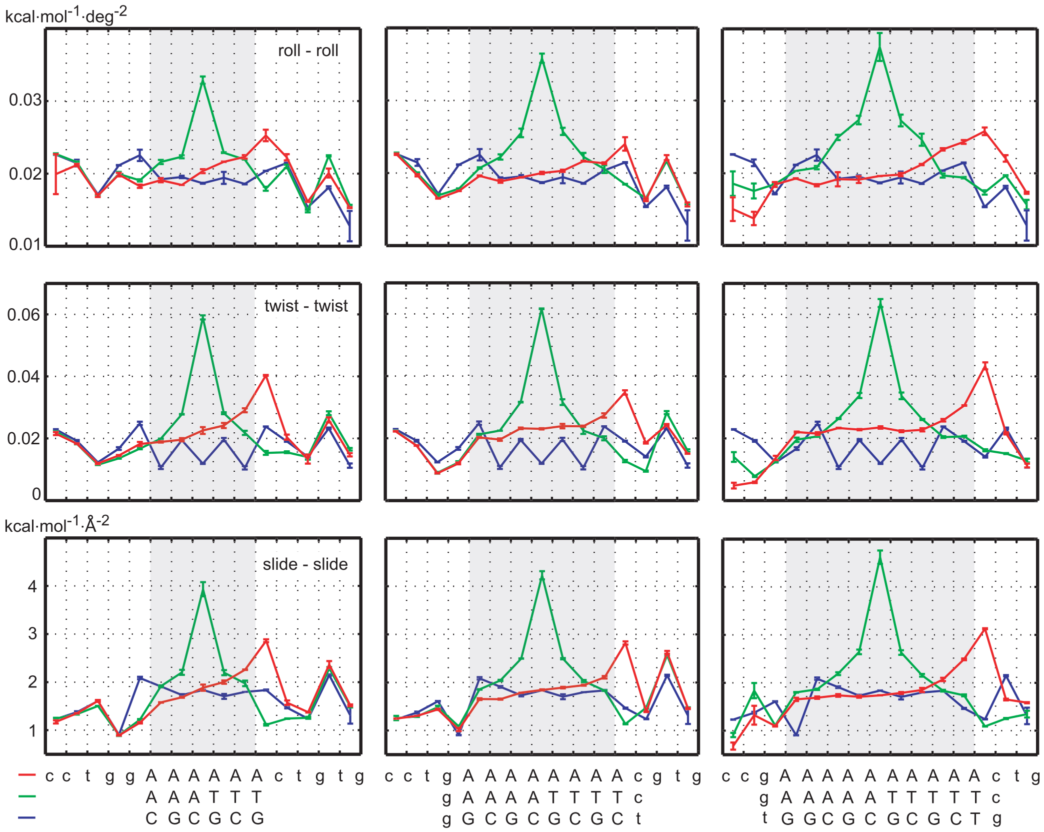

Figure S2: Unidimensional stiffness constants defined by eq (9) in the main text for roll, twist and slide.

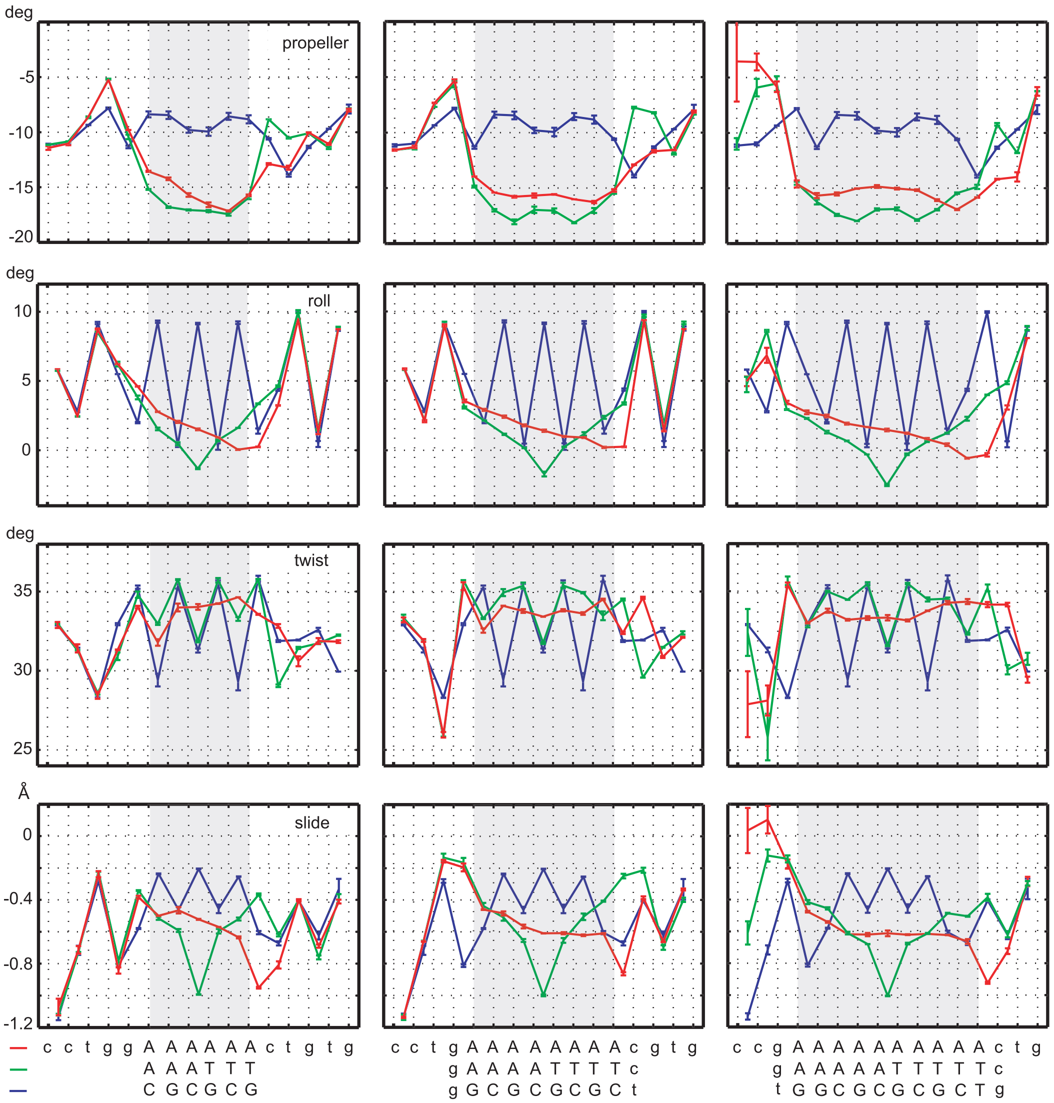

Figure S3: Equilibrium values of propeller, roll, twist and slide.

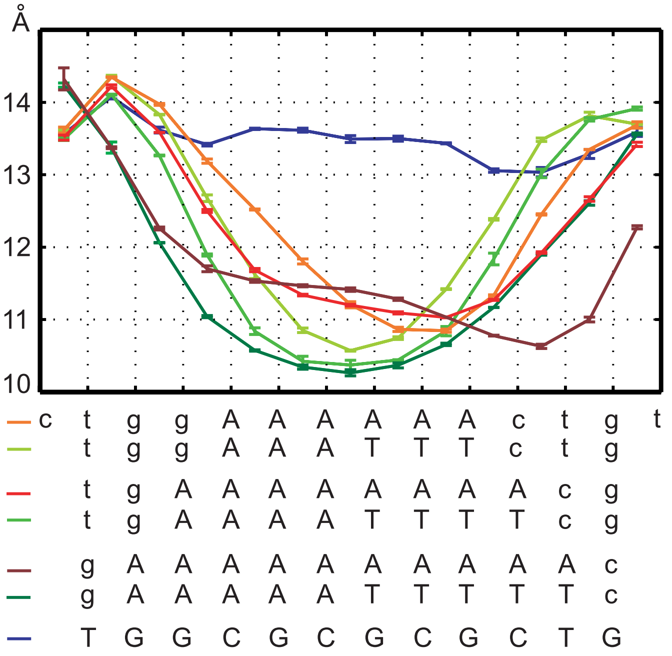

Figure S4: Minor groove widths.

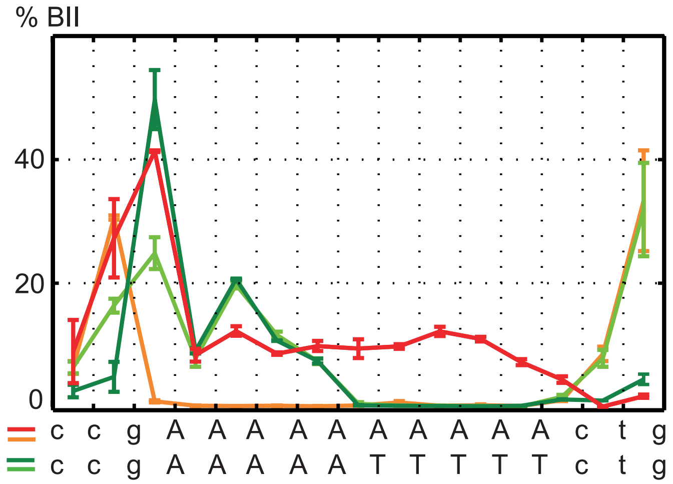

Figure S5: Populations of BII substates. Values for the reference strand (indicated) are in dark colour, values for the complementary strand are in light colour. Both strands are taken in the 5' to 3' direction, so that values for the two strands at each position should coincide for palindromic sequences.

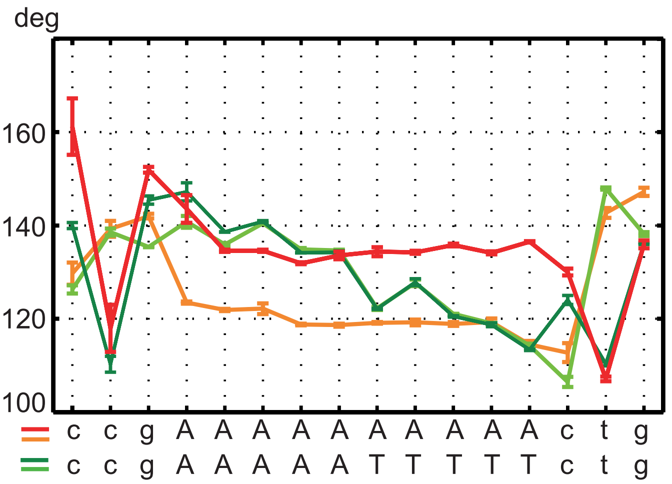

Figure S6: Mean values of sugar pucker. Colour coding as in Figure S5.

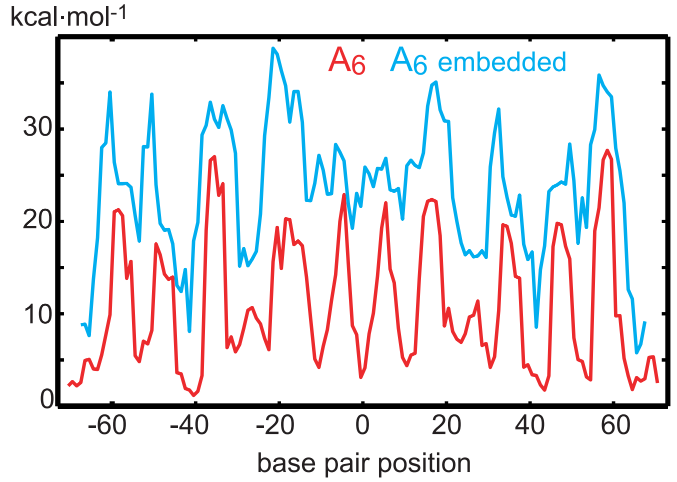

Figure S7: Energy profiles due to threading of isolated A_6 tract (red) and A_6 tract together with its flanking sequences (12 bp in total, see Table 1 of the main text - cyan). The energy is higher for the embedded tract, since the sequence is longer. However, the phasing of the periodic profile is very similar.

A3T3					
66.8	(1.2)	2.0	(1.9)	15.2	(3.7)
2.0	(1.9)	137.3	(1.0)	-2.6	(0.7)
15.2	(3.7)	-2.6	(0.7)	121.4	(2.6)
A6					
53.2	(0.0)	-2.4	(0.6)	21.2	(0.1)
-2.4	(0.6)	118.2	(0.9)	-1.5	(1.1)
21.2	(0.1)	-1.5	(1.1)	110.5	(0.4)
A4T4					
91.4	(0.7)	0.7	(2.4)	11.0	(0.9)
0.7	(2.4)	93.6	(3.1)	-0.7	(3.9)
11.0	(0.9)	-0.7	(3.9)	136.3	(0.8)
A8					
67.6	(1.6)	0.3	(0.4)	29.4	(0.2)
0.3	(0.4)	81.0	(0.3)	12.9	(1.4)
29.4	(0.2)	12.9	(1.4)	115.5	(1.6)
A5T5					
113.7	(6.4)	1.7	(2.8)	3.7	(4.7)
1.7	(2.8)	75.0	(2.8)	0.2	(3.1)
3.7	(4.7)	0.2	(3.1)	149.8	(4.5)
A10					
89.2	(2.1)	1.9	(4.0)	28.8	(2.2)
1.9	(4.0)	61.5	(2.8)	9.9	(0.4)
28.8	(2.2)	9.9	(0.4)	123.8	(2.8)
CG6					
65.5	(1.5)	0.6	(1.2)	36.1	(1.1)
0.6	(1.2)	112.3	(2.0)	1.6	(0.4)
36.1	(1.1)	1.6	(0.4)	137.3	(3.2)
CG8					
73.8	(2.9)	-0.1	(0.4)	25.4	(2.3)
-0.1	(0.4)	77.0	(1.2)	2.0	(0.9)
25.4	(2.3)	2.0	(0.9)	115.0	(2.1)
CG10					
88.4	(1.9)	-0.7	(0.2)	24.3	(0.9)
-0.7	(0.2)	66.9	(0.9)	1.4	(1.3)
24.3	(0.9)	1.4	(1.3)	121.9	(1.6)

Table S1: Stiffness matrices for the elastic rod model of A-tracts alone (without flanking sequences). Matrices for the corresponding subsequences of the control sequence are also shown (CGm - m central base pairs of the control). Coordinates are ordered as follows: global roll, global tilt, total twist. Errors are mean differences between values for the whole trajectory and for its halves.

Sequence	Data source	Bending nagnitude (°)	Bending direction (°)
A_6	MD	4.7 (0)	18 (1)
	1fzx	7.1 (0)	138 (2)
	bdl047/I	5.6	171
	bdl047/II	4.9	173
	bdl047/III	4.2	181
A_3T_3	MD	4.2 (0)	3 (2)
	bd0067	4.1	255
	bdl038	11.8	341
A_8	MD	5.2 (0)	355 (1)
A_4T_4	MD	5.4 (0)	2 (1)
A_{10}	MD	5.4 (0)	322 (5)
A_5T_5	MD	2.5 (0)	355 (0)
CG6	MD	15 (0)	360 (0)
CG8	MD	18 (0)	359 (0)
CG10	MD	15 (0)	358 (1)

Table S2: Bending magnitude and direction of the A-tracts alone (without flanking sequences) and the corresponding parts of the control. The direction of 0° indicates bending towards the major groove, 180° means bending towards the minor groove. Errors are mean differences between values for the whole trajectory and for its halves (MD) or standard deviations over the NMR models (1fzx).
